# Pathological complete response following neoadjuvant chemotherapy for locally advanced intrahepatic cholangiocarcinoma

**DOI:** 10.1186/s40792-024-01832-x

**Published:** 2024-02-09

**Authors:** Yoshitaka Shimamaki, Isamu Hosokawa, Tsukasa Takayashiki, Shigetsugu Takano, Itaru Sonoda, Masayuki Ohtsuka

**Affiliations:** Department of General Surgery, Chiba University Graduate School of Medicine, 1-8-1 Inohana, Chuo-Ku, Chiba, 260-0856 Japan

**Keywords:** Intrahepatic cholangiocarcinoma, Neoadjuvant chemotherapy, Pathological complete response

## Abstract

**Background:**

Intrahepatic cholangiocarcinoma (ICC) is the second most common primary liver cancer. Cases when found are often advanced with vascular invasion, and radical resection is often difficult. Despite curative resection, the postoperative recurrence rate of patients with histological lymph node metastasis is high, and their prognosis is poor. Therefore, there is an urgent need to establish multidisciplinary treatment that combines chemotherapy and surgical resection. The efficacy of neoadjuvant chemotherapy (NAC) for locally advanced ICC is unclear. In this report, a case of locally advanced ICC in which pathological complete response (pCR) was achieved after NAC is described.

**Case presentation:**

A 79-year-old woman was admitted to a local hospital with appetite loss. Computed tomography showed a 100 × 90 mm low-contrast tumor in the left hepatic lobe and segment 1 with invasion to the inferior vena cava (IVC), and several lymph nodes along the left gastric artery and lesser curvature were enlarged. Therefore, she was treated with a combined chemotherapy regimen of gemcitabine and cisplatin. After four courses, the tumor size decreased to 30 × 60 mm without invasion to the IVC. Left hepatectomy extending to segment 1 with bile duct resection combined with middle hepatic vein resection (H1234-B-MHV), dissection of regional lymph nodes and pyloroplasty were performed. After radical resection, pCR was achieved. She is alive with no evidence of disease, 2 years after surgery.

**Conclusions:**

In this case, a patient with locally advanced ICC achieved pCR to NAC. NAC may be effective for ICC. Patients who achieve pCR may have a better prognosis.

## Background

Intrahepatic cholangiocarcinoma (ICC) arising from the epithelial cells of the intrahepatic bile ducts is the second most common, but still relatively rare, primary hepatic tumor. Surgical resection is the only curative treatment [1]. Since it is difficult to identify early ICC, when it is found, it is often advanced with vascular invasion and lymph node metastasis, and radical resection is difficult. Even if it is possible to achieve curative resection, the postoperative recurrence rate and metastasis rate are high, and the prognosis is poor. Therefore, there is an urgent need to establish multidisciplinary treatment that combines chemotherapy and surgical resection.

Currently, gemcitabine (GEM) plus cisplatin (CDDP) has become the standard first-line regimen for the treatment of patients with unresectable or metastatic biliary tract cancer (BTC) including ICC [2]. However, there is no evidence of the efficacy of neoadjuvant chemotherapy (NAC) for potentially resectable ICC.

In this report, a case of locally advanced ICC that achieved pathological complete response (pCR) after NAC is described.

## Case presentation

A 79-year-old woman with loss of appetite was transferred from another hospital. On physical examination at the first visit, no abdominal tenderness and jaundice were noted. Initial laboratory findings were unremarkable, except for a high level of carbohydrate antigen 19–9 (CA19-9; 125.7 U/ml). Both serum HBs-antigen and HCV-antibody tests were negative.

**Fig. 1:**
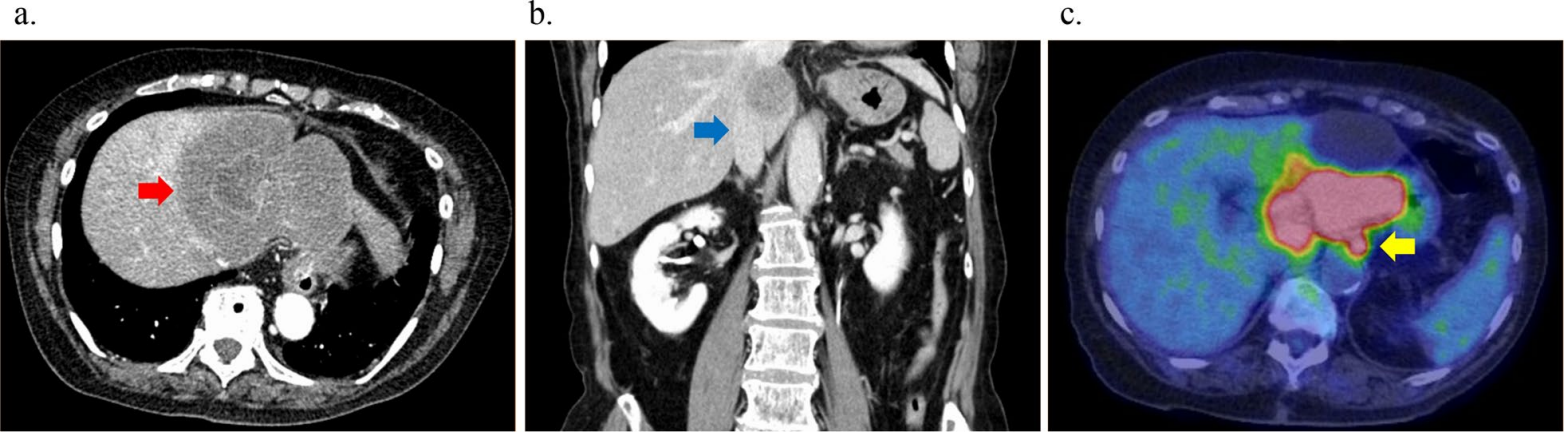
Radiological examinations before neoadjuvant chemotherapy (NAC). **a**, **b** Computed tomography (CT) shows a 100-mm tumor in the left hepatic lobe and segment 1. The tumor has invaded to the inferior vena cava (IVC) (**a** axial view of CT; **b** Coronal view of CT). **c** Positron emission tomography (PET) shows fluorodeoxyglucose (FDG) uptake around the lesser curvature nodes

Abdominal computed tomography (CT) showed a 100 × 90 mm low-contrast tumor in segments 1, 2, 3, and 4 resulting in dilated bile ducts in B2, enlarged regional lymph nodes #3 and #7 [#3 and #7, according to the General Rules for Clinical and Pathological Studies on Primary Liver Cancer], and invasion of the inferior vena cava (IVC), left portal vein (LPV), middle hepatic vein (MHV), and middle hepatic artery (MHA) (Fig. 1a, b).

Positron emission tomography with ^18^F-fluoro-D-deoxy-glucose (^18^F-FDG PET)/CT showed uptake by the primary liver tumor and regional lymph nodes (Fig. 1c). On imaging, the patient was diagnosed with intrahepatic cholangiocarcinoma (ICC) with regional lymph node metastases [cT2N1M0, cStage IIIB according to the Union for International Cancer Control (UICC) classification system].

Radical surgical resection including combined vascular resection, reconstruction, and lymph node dissection was possible, but R0 resection is often difficult in cases of locally advanced ICC such as the present case; thus, it was not considered achievable during discussion in the multidisciplinary team (MDT) meeting. It was also thought that her prognosis would be poor due to inability to achieve R0 resection. Thus, she was treated with combined chemotherapy due to the lymph node metastases. The combination chemotherapy consisted of gemcitabine (1000 mg/m^2^) plus cisplatin (25 mg/m^2^) administered for 2 weeks followed by a 1-week respite, with a single course extending over 3 weeks.

**Fig. 2:**
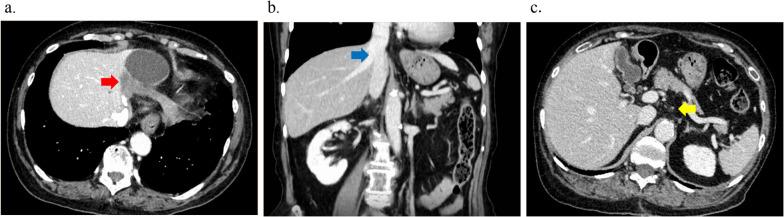
Radiological examinations after neoadjuvant chemotherapy (NAC). **a**, **b** The size of the tumor has decreased to 18 mm, and the tumor is no longer attached to the IVC (**a** axial view of CT; **b** coronal view of CT). **c** Lymph node enlargement has almost disappeared (axial view of CT)

**Fig. 3:**
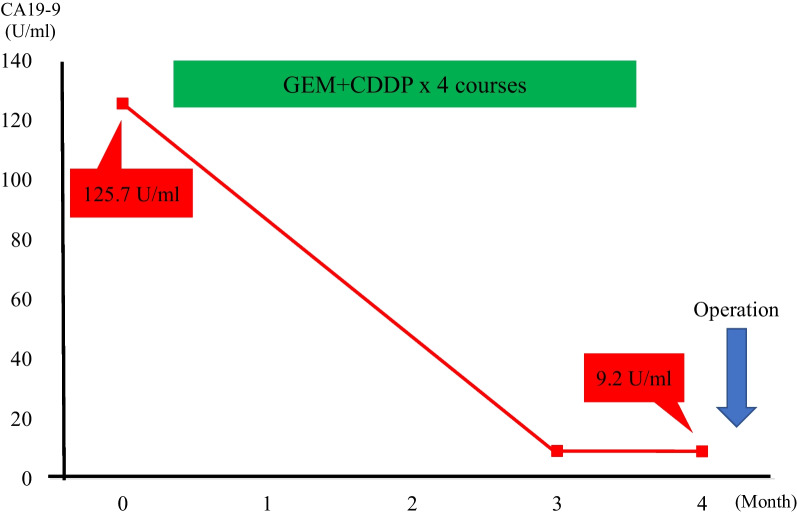
Serum carbohydrate antigen 19–9 (CA19-9) levels during NAC

After 4 courses of combination chemotherapy without adverse events, CT showed a marked reduction of the primary tumor and metastatic lymph nodes. The size of the primary tumor had decreased to 30 × 60 mm, and the invasion of the IVC, LPV, MHV, and MHA had disappeared (Fig. 2a–c). The lymph node enlargement also disappeared, and the CA19-9 level decreased to within the normal range (9.3 U/ml) (Fig. 3). The patient achieved a partial response (PR) as assessed using Response Evaluation Criteria In Solid Tumor (RECIST), and the post-chemotherapy TNM staging was ycT2N0M0 Stage II by the UICC criteria. At the MDT meeting, it was then decided that surgery could proceed. H1234-B-MHV, dissection of #1, #3, #5, and #7 lymph nodes, and pyloroplasty to prevent the delayed excretion of gastric contents due to the vagus nerve reflex associated with lymph node dissection were performed. She was discharged on postoperative day 15.

**Fig. 4:**
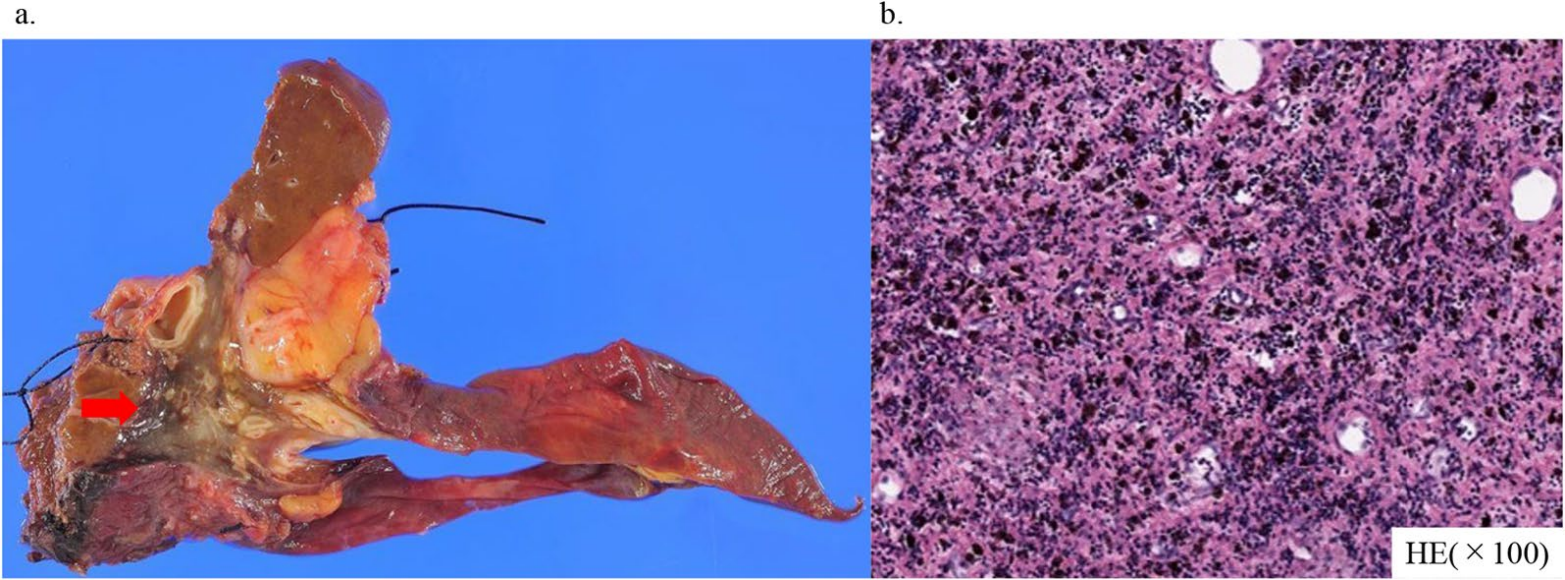
Pathological findings. **a** Specimen of left hemi-hepatectomy including the MHV and S1. The brown and soft tumor is 26 mm in the center of the specimen. **b** Microscopic pathological examination of the surgical specimen shows fibrosis, invasion of inflammatory cells, and macrophages phagocytosing hemosiderin. No viable tumor is present

The histological examination showed scattered pigmented macrophages in the fibrotic tissue and inflammatory cell infiltration. No invasive carcinoma or epithelial carcinoma components were found. There were no viable carcinoma cells in the dissected lymph nodes (Fig. 4). The pathological TNM staging was T0N0M0 according to the UICC criteria. In the MDT meeting, the decision was made not to proceed with adjuvant chemotherapy, for the reason that pCR had been achieved.

She remains alive with no evidence of disease 2 years after surgery without adjuvant chemotherapy.

## Discussion

The case of a patient with locally advanced ICC at presentation who achieved a pathological complete response after undergoing NAC following surgical resection was described.

Most patients with ICC are diagnosed at an advanced stage due to the lack of abdominal symptoms. Surgical resection currently represents the only potentially curative treatment for ICC. Therefore, surgical resection should be performed with a negative pathological margin (R0) and preservation of adequate future remnant liver. In surgical resection, R0 resection is one of the best prognostic factors [3]. However, the prognosis for patients with ICC after resection remains unsatisfactory. Si et al. evaluated 251 patients who underwent curative resection; 165 of these patients developed recurrence within 5 years, and the 5-year survival rate was 32.3% [4].

Lymph node metastasis has been associated with a poor prognosis. A multi-institutional analysis of 449 patients for whom radical resection was performed for ICC concluded that patients with lymph node metastases had a worse outcome (median survival time with N0, 30 months vs N1, 24 months; *P* = 0.03) [5]. Furthermore, the number of lymph node metastases was a prognostic factor in ICC. Patients with more than three lymph node metastases had worse outcomes than patients with one or two lymph node metastases (3-year overall survival: 0% vs. 50%) [6].

The gemcitabine plus cisplatin (GC) regimen has become the standard for the treatment of patients with unresectable or metastatic BTC including ICC. The GC regimen based on the ABC-02 phase III trial in the United Kingdom showed a significant advantage of GC therapy in median overall survival compared to GEM alone (11.7 vs. 8.1 months, *P* < 0.001) [2]. In Japan, the BT-22 trial used the same regimen as the ABC-02 trial and showed the efficacy and safety of GC therapy [7]. Recently, the KHBO1401-MITSUBA trial, a randomized phase III trial, showed the efficacy of GC plus S-1 regimen. Patients with advanced BTC who developed recurrence after surgery, or for whom curative surgery was not an option (unresectability was determined at the discretion of each institution) received GCS therapy, which consisted of intravenous gemcitabine (1000 mg/m^2^) and cisplatin (25 mg/m^2^) on day 1 and oral administration of S-1 (80 mg/m^2^) on days 1–7, every 2 weeks. Median OS and the 1-year OS rate were 13.5 months and 59.4% in the GCS arm and 12.6 months and 53.7% in the GC arm, respectively (*P* = 0.046) [8].

Conversion surgery is defined as standard resection performed for previously unresectable cases that become resectable as a result of regression following chemotherapy. In cases of intrahepatic cholangiocarcinoma, it has been reported that conversion surgery was possible in 17.3% of cases after tumor shrinkage had been achieved [9]. According to the criteria of Kato, our case was considered unresectable [10], and NAC was administered in anticipation of surgery.

The efficacy of NAC for potentially resectable ICC remains unknown, with limited reports showing the efficacy of NAC. Sutton et al. considered 52 cases of resectable ICC, 10 of which received NAC; NAC was independently associated with improved overall survival (HR = 0.16, *P* = 0.01) [11]. However, since this was a retrospective study, prospective trials should be performed to determine the efficacy of NAC for resectable ICC.

Pathological complete response to preoperative chemotherapy is rare for ICC, with only five cases reported (Table 1) [10, 12–15]. The rate of pCR to chemotherapy for ICC is currently unknown. All cases had distant metastases or locally advanced lymph node metastases. There was no similar pathological feature. There were no similar pathological features among the cases. In our case, scattered pigmented macrophages were observed in fibrotic tissue with inflammatory cell infiltration. Phagocytosing cancer cells destroyed by chemotherapy were apparent as scattered pigmented macrophages. All cases achieved survival of more than 6 months. A pathological complete response after NAC may be associated with a better prognosis. In pancreatic cancer and colorectal liver metastases, the response rate to preoperative chemotherapy has already been found to be associated with a better prognosis [16, 17].

**Table 1:** Details of previous reports

Case	Age (years), Sex	Preoperative chemotherapy	Operative procedures	Pathological features	Survival
1	33, male	Doxorubicin, cisplatin, 5-FU, IFN K (9 courses)	Right hepatectomy	Necrosis	> 30 months
2	67, male	GEM + oxaliplatin (4 courses) GEM + cisplatin (5 courses)	Left trisectionectomy caudate lobectomy	Calcification	> 6 months
3	60, male	GEM + cisplatin (4 courses)	Left trisectionectomy caudate lobectomy	Fibrosis	> 7 years
4	72, male	GEM + S-1 (10 courses)	Left hepatectomy	Necrosis	> 6 months
5	47, male	GEM + cisplatin + nabPTX (3 courses) Pembrolizumab (3 months)	Left hepatectomy	Necrosis, fibrosis Chronic inflammation	> 6 months
Present case	79, female	GEM + cisplatin (4 courses)	Left hepatectomy caudate lobectomy	Fibrosis, macrophages phagocytosing hemosiderin	> 6 months

IFN K: Interferon alpha Kinoid

As for ICC, even in cases of advanced ICC with lymph node metastases, cases that achieve pCR may achieve long-term survival.

## Conclusion

A case of locally advanced ICC that achieved pCR to NAC was described. The association of pathological response with prognosis should be considered. Further evidence and prospective studies are required to evaluate the efficacy of NAC for ICC.

## Data Availability

Data sharing is applicable to this article.

## Abbreviations

ICC: Intrahepatic cholangiocarcinoma
GEM: Gemcitabine
GC: Gemcitabine plus cisplatin
BTC: Biliary tract cancer
NAC: Neoadjuvant chemotherapy
pCR: Pathological complete response
CA19-9: Carbohydrate antigen 19–9
IVC: Inferior vena cava
LPV: Left portal vein
MHV: Middle hepatic vein
MHA: Middle hepatic artery
PET: Positron emission tomography
FDG: Fluorodeoxyglucose
UICC: Union for International Cancer Control
MDT: Multidisciplinary team
